# Meta-analysis of epigenetic aging in schizophrenia reveals multifaceted relationships with age, sex, illness duration, and polygenic risk

**DOI:** 10.1186/s13148-024-01660-8

**Published:** 2024-04-08

**Authors:** Anil P. S. Ori, Loes M. Olde Loohuis, Jerry Guintivano, Eilis Hannon, Emma Dempster, David St. Clair, Nick J. Bass, Andrew McQuillin, Jonathan Mill, Patrick F. Sullivan, Rene S. Kahn, Steve Horvath, Roel A. Ophoff

**Affiliations:** 1 Center for Neurobehavioral Genetics, Semel Institute for Neuroscience and Human Behavior, University of California Los Angeles, Gonda Center, Room 4357B, 695 Charles E. Young Drive South, Los Angeles, CA 90095-176 USA; 2 Present Address: Department of Psychiatry, Amsterdam UMC, University of Amsterdam, Amsterdam, The Netherlands; 3 Department of Genetics, University of North Carolina, Chapel Hill, NC USA; 4 University of Exeter Medical School, University of Exeter, Exeter, UK; 5 Institute of Medical Sciences, University of Aberdeen, Aberdeen, Scotland, UK; 6 Division of Psychiatry, University College London, London, UK; 7 Department of Medical Epidemiology and Biostatistics, Karolinska Institutet, Stockholm, Sweden; 8 Icahn School of Medicine at Mount Sinai, Department of Psychiatry, New York, NY USA; 9 Department of Biostatistics, Fielding School of Public Health, University of California Los Angeles, Los Angeles, CA USA; 10 Department of Human Genetics, David Geffen School of Medicine, University of California Los Angeles, Los Angeles, CA USA; 11 Department of Psychiatry, Erasmus University Medical Center, Rotterdam, The Netherlands

**Keywords:** Schizophrenia, DNA methylation, Aging, Epigenetic clocks, Biological aging, Accelerated aging, Polygenic risk, Mortality risk

## Abstract

**Background:**

The study of biological age acceleration may help identify at-risk individuals and reduce the rising global burden of age-related diseases. Using DNA methylation (DNAm) clocks, we investigated biological aging in schizophrenia (SCZ), a mental illness that is associated with an increased prevalence of age-related disabilities and morbidities. In a whole blood DNAm sample of 1090 SCZ cases and 1206 controls across four European cohorts, we performed a meta-analysis of differential aging using three DNAm clocks (i.e., Hannum, Horvath, and Levine). To dissect how DNAm aging contributes to SCZ, we integrated information on duration of illness and SCZ polygenic risk, as well as stratified our analyses by chronological age and biological sex.

**Results:**

We found that blood-based DNAm aging is significantly altered in SCZ independent from duration of the illness since onset. We observed sex-specific and nonlinear age effects that differed between clocks and point to possible distinct age windows of altered aging in SCZ. Most notably, intrinsic cellular age (Horvath clock) is decelerated in SCZ cases in young adulthood, while phenotypic age (Levine clock) is accelerated in later adulthood compared to controls. Accelerated phenotypic aging was most pronounced in women with SCZ carrying a high polygenic burden with an age acceleration of + 3.82 years (CI 2.02–5.61, *P* = 1.1E−03). Phenotypic aging and SCZ polygenic risk contributed additively to the illness and together explained up to 14.38% of the variance in disease status.

**Conclusions:**

Our study contributes to the growing body of evidence of altered DNAm aging in SCZ and points to intrinsic age deceleration in younger adulthood and phenotypic age acceleration in later adulthood in SCZ. Since increased phenotypic age is associated with increased risk of all-cause mortality, our findings indicate that specific and identifiable patient groups are at increased mortality risk as measured by the Levine clock. Our study did not find that DNAm aging could be explained by the duration of illness of patients, but we did observe age- and sex-specific effects that warrant further investigation. Finally, our results show that combining genetic and epigenetic predictors can improve predictions of disease outcomes and may help with disease management in schizophrenia.

**Supplementary Information:**

The online version contains supplementary material available at 10.1186/s13148-024-01660-8.

## Introduction

As the population continues to age, reducing the burden of age-related disability and morbidity is timely and important, particularly for mental illnesses [1, 2]. Ranked as one of the most disabling illnesses globally [3], schizophrenia (SCZ) has significant impact on patients, families, and society. SCZ is associated with a two- to threefold increased risk of mortality [4–6] and a 15-year reduction in life expectancy compared to the general population [7, 8]. Despite elevated rates of suicide and other unnatural causes of death, most morbidity in SCZ is attributed to age-related diseases, such as cardiovascular and respiratory diseases and diabetes mellitus [5, 9, 10]. Processes of biological aging may therefore be accelerated in patients diagnosed with SCZ, either through an increased prevalence of age-related conditions or as a more integrated part of the illness [11]. Quantification of biological aging can help with identification of at-risk individuals or even prevention of age-related diseases [12, 13]. Various age-related biomarkers have been shown to have significantly deviating levels in people diagnosed with SCZ compared to controls [14], which may offer new opportunities to study the phenomenon of biological age in SCZ.

DNAm age predictors, or “epigenetic clocks,” are biomarkers of aging that generate a highly accurate estimate of chronological age, known as DNAm age [15–17]. The difference between predicted DNAm and chronological age (Δage) is associated with a wide-range of health and disease outcomes, including all-cause mortality [18–21], socioeconomic adversity and smoking [22], metabolic outcomes, such as body mass index (BMI) and obesity [23, 24], and brain-related phenotypes, such as Parkinson's disease, posttraumatic stress disorder, insomnia, major depressive disorder, and bipolar disorder [25–29]. As epigenetic signatures can be modifiable [30], DNAm-based predictors hold great promise for clinical utilization.

While first reports on DNAm age in SCZ found limited to no evidence for altered biological age in either brain or blood in SCZ [31–34], recent larger studies reported both DNAm age acceleration in whole blood and age deceleration in patients compared to controls with evidence of age-specific effects [35, 36]. These studies analyzed multiple cohorts, but either analyzed cohorts separately or did not take into account between-cohort sampling variability, which makes it difficult to interpret their findings as results may have been biased by a single study. Meta-analyses can statistically combine results across cohorts, weight effect sizes by cohort sample size, and evaluate heterogeneity between studies, which allows for a more precise quantification and decreases the probability of false negative results [37]. Furthermore, as age-related conditions and morbidities in the SCZ population differ between older and younger individuals, and women and men [5], age- and sex-stratified analyses of DNAm aging are warranted as well, as these can provide important insights on how biological age is impacted in SCZ that would otherwise be missed. Finally, it is unknown how illness duration and polygenic risk are associated with DNAm age. It may be that adverse effects of the illness compound over time and result in accelerated biological age in patients. On the other hand, mechanisms of aging may be more intrinsically altered in SCZ.

To investigate DNAm age in SCZ, we used three independent DNAm age estimators: the Hannum [16], Horvath [15], and Levine clock [17]. These clocks were also included in previous studies of DNAm age in SCZ. Each predictor is designed using different training features and captures distinct characteristics of aging [38]: (i) the Hannum age predictor was trained on whole blood adult samples, (ii) the Horvath predictor was trained across 30 tissues and cell types across developmental stages, and (iii) the Levine combines DNAm from adult blood samples with clinical blood-based measures. As the Levine estimator is trained on chronological age and nine clinical markers, its output is referred to as DNAm PhenoAge or “phenotypic age.” The Hannum estimator is said to capture cell extrinsic aging in blood, whereas the Horvath clock measures more cell intrinsic aging as it was trained across multiple tissues and therefore is less dependent on cell type composition. All three clocks, in different but complementary ways, capture the pace of biological aging that is associated with various age-related conditions and diseases, including all-cause mortality [19, 38].

Here, we implemented these three DNAm clocks across four European case–control cohorts, representing one of the largest analyses of DNAm age in SCZ so far. We performed a meta-analysis across the full sample to obtain precise effect size estimates for each clock and evaluated heterogeneity between cohorts. We also stratified analyses by age and sex and integrated DNAm age with duration of illness and SCZ polygenic risk in a subsample of our cohort to further investigate how DNAm aging is impacted in the illness. DNAm smoking scores and blood cell type proportions were used to gain further insights into differential aging patterns. This study reports an in-depth investigation of the DNAm aging landscape in schizophrenia.

## Results

**Fig. 1:**
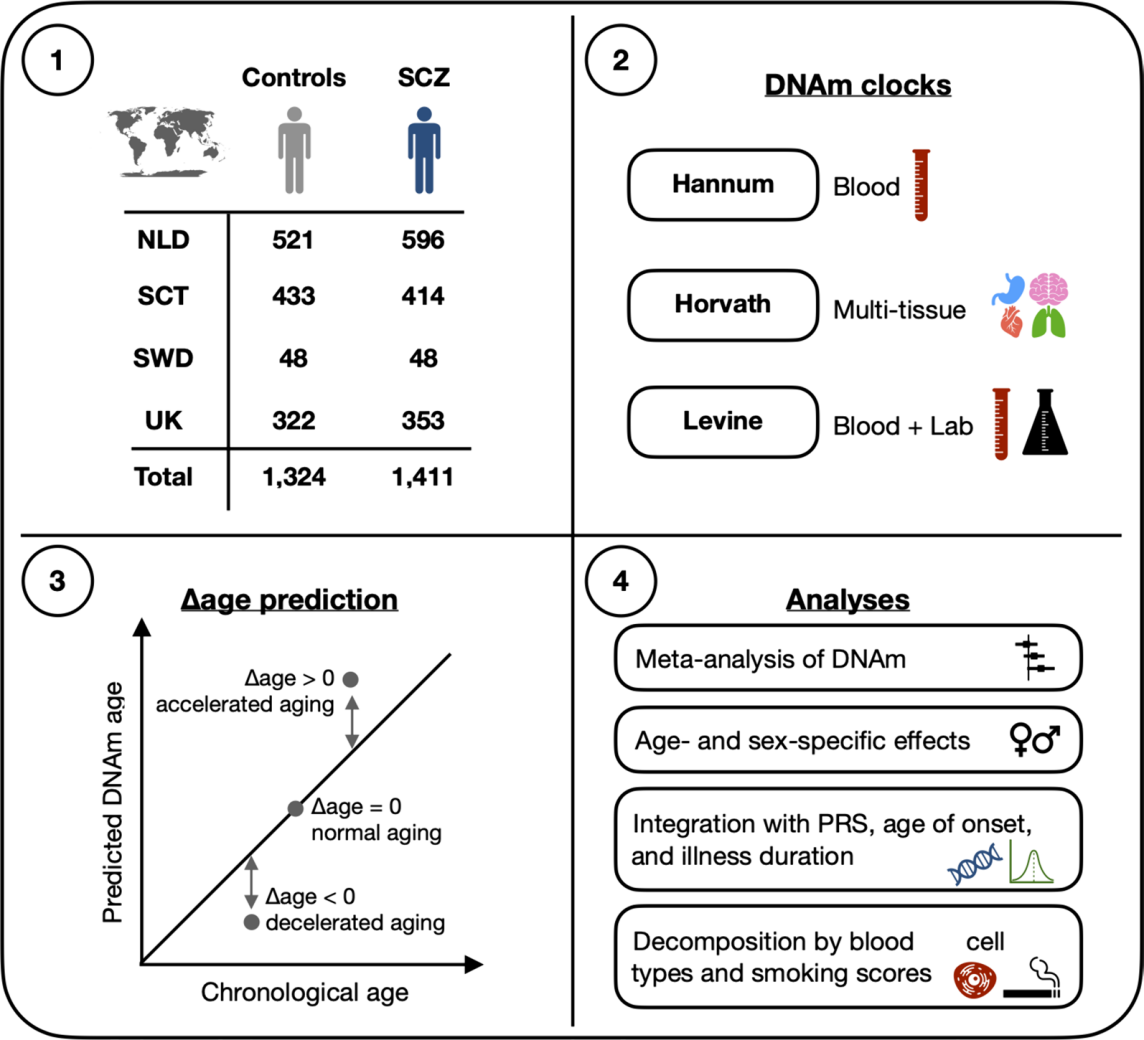
Overview of study design and analysis framework. DNA methylation (DNAm) data were available for a total of 2735 samples across four European cohorts. See Additional file 1: Table S2 for more details on samples. DNAm age estimates were generated using three DNAm clocks, each designed to capture different features of aging (box 2). To investigate differences in aging between cases and controls, Δage was computed (box 3) and analyzed according to the stepwise framework shown in box 4. SCZ, schizophrenia; NLD, the Netherlands; SCT, Scotland; SWD, Sweden; UK, United Kingdom; PRS, polygenic risk scores

Figure 1 shows a schematic overview of the study design and analysis framework used to investigate DNAm aging in SCZ. After data preprocessing and quality control, 1090 SCZ cases and 1206 controls (2296 subjects of 2707 initial samples) were included in our analysis. The overall sample has a mean age of 40.3 years (SD = 14.4) and consists of 34.5% women (Additional file 1: Table S1 and Additional file 2: Fig. S1).

**Fig. 2:**
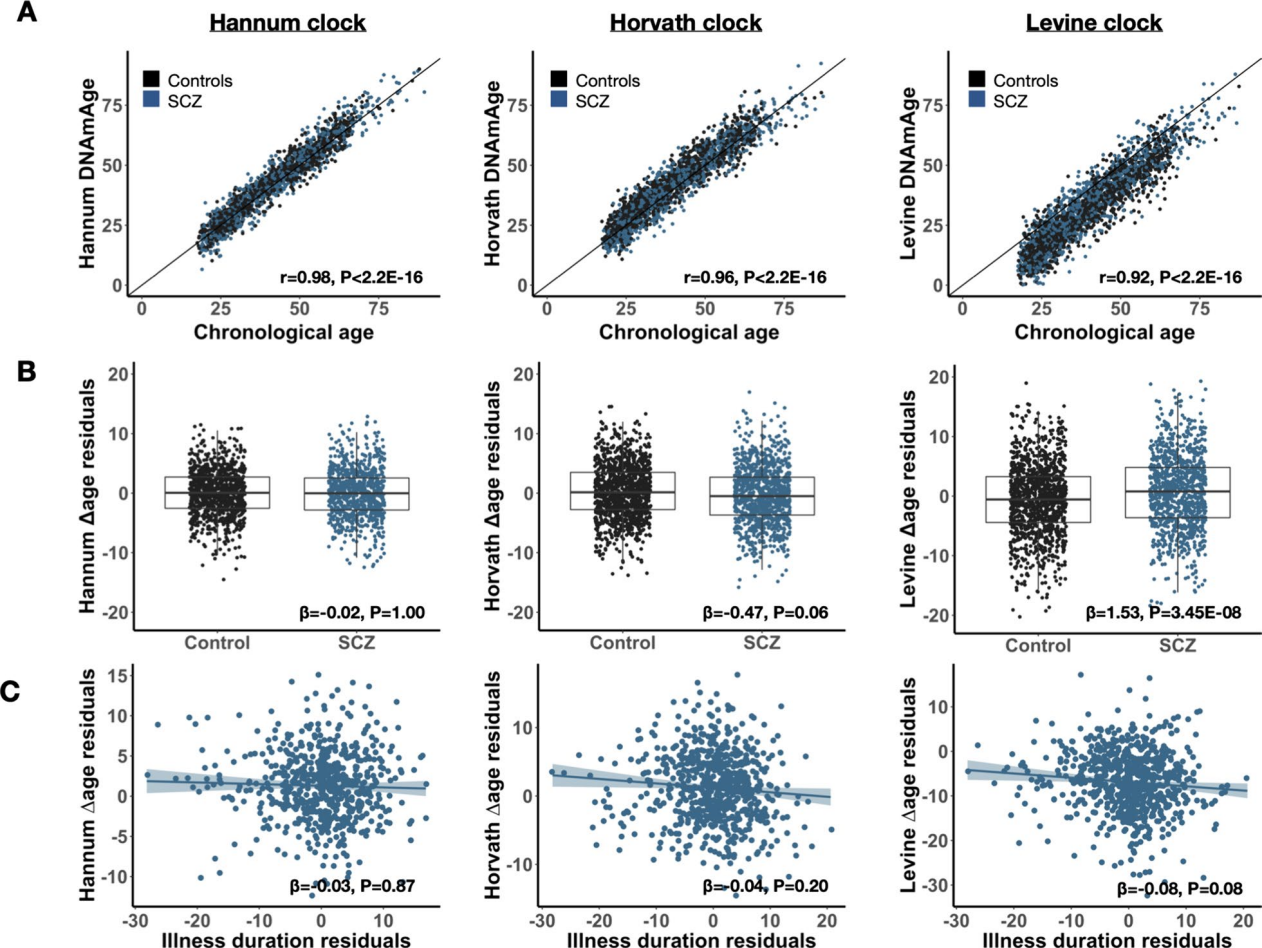
DNA methylation aging is altered in schizophrenia independent from illness duration. Presented are results visualizing DNAm aging in SCZ for each clock: Hannum (left), Horvath (middle), and Levine (right). Cases are shown in blue and controls in black. **A** Correlation between DNAm age and chronological age. The Pearson’s correlation estimates and corresponding p-values are shown in the bottom corner. **B** Boxplots of Δage between cases and controls. β represents the mean change in Δage in cases compared to controls. **C** Relationship between Δage and illness duration with a regression line fitted. Illness duration was adjusted for cohort, age, and sex and the residuals plotted. β represents the change in Δage in cases per year of chronological age compared to controls. In (**B**–**C**), the meta-analytic coefficients and p-values are shown. All *P*-values are adjusted for multiple testing across clocks (*n* = 3)

Across cohorts, all three clocks produce a high correlation with chronological age (Pearson’s *r* = 0.92–0.94; Fig. 2A and Additional file 2: S2). Using duplicates in the Dutch cohort, we assessed consistency between pairs of technical replicates, i.e., samples for which blood was collected at the same time, but DNA processed at different times and DNAm data obtained on different arrays. Comparing Δage estimates between these pairs, we find a significant correlation for each clock (Additional file 2: Fig. S3): Hannum (rho = 0.79, *n* = 10), Horvath (rho = 0.53, *n* = 118), and Levine (rho = 0.67, *n* = 118). Δage directionality (i.e., age deceleration or acceleration) is concordant in 90%, 73%, and 86% of pairs for Hannum, Horvath, and Levine, respectively, highlighting that the obtained estimates of DNAm age are reproduced across all three clocks. Comparing Δage estimates between clocks using all samples, we find a moderate concordance (Pearson’s *r* = 0.39–0.43; Additional file 2: Fig. S4), demonstrating that a significant proportion of the variation in Δage is clock-specific. As these three estimators were trained on different features of biological aging, investigating them in conjunction may thus yield broader insights into differential aging.

### DNA methylation age is altered in SCZ independent from duration of illness

Across the full sample, patients with SCZ are on average 1.53 years older in phenotypic Δage (Levine clock) compared to controls (P_meta_ = 3.45E−08) (Fig. 2B). The intrinsic cellular age (Horvath) predictor revealed an opposite pattern, with SCZ cases appearing 0.47 years younger compared to controls (P_meta_ = 0.06). No differences were observed between cases and controls when applying the blood-based Hannum DNAm age predictor (*β* = − 0.02, P_meta_ = 1.00). There was no heterogeneity between the four cohorts (P_het_ > 0.05, Additional File 3: Table S5). We found no association between DNAm aging and duration of illness in SCZ patients for any of the three epigenetic clocks, nor did we for age of onset (Fig. 2C, Additional file 3: Table S6).

### Age- and sex-specific effects contribute to DNAm aging

To investigate whether DNAm aging in SCZ differed by age and sex, we first quantified the overall contribution of the interaction of age and sex with disease status on Δage in the combined sample. For the Horvath and Levine clock, inclusion of an interaction term between age and case–control status presented a significantly better model fit, with the three-way interaction model (i.e., disease status, age and sex) explaining the most variance in Δage (Table 1 and Additional file 1: S7). The three-way interaction was a significant improvement of model fit for Levine Δage (*P* = 0.02), but not for Horvath Δage (*P* = 0.34), suggesting that sex-specific effects are more pronounced for Levine Δage. We found no evidence of age- and sex-specific effects for Hannum Δage.

**Table 1:** Age- and sex-specific effects significantly contribute to DNAm aging in schizophrenia

		Hannum Δage		Horvath Δage		Levine Δage	
Model variables	Comparison	*R*^2^ (%)	*P*-value	*R*^2^ (%)	*P*-value	*R*^2^ (%)	*P*-value
Model 0: baseline	–	6.9	–	3.6	–	2.1	–
Model 1: + status	Model 0 vs. 1	6.9	1.00	4.0	9.8E−03	3.2	6.7E−06
Model 2: + status*age.cont	Model 1 vs. 2	7.1	0.34	4.3	0.13	3.7	5.3E−03
Model 3: + status*age.groups	Model 1 vs. 3	7.4	0.24	5.5	2.0E−05	4.0	0.02
Model 4: + status*age.groups*sex	Model 3 vs. 4	7.7	1.00	5.9	0.34	4.7	0.02

Shown are the contributions of interaction effects between disease status and age and sex on Δage. The baseline model corresponds to Δage ~ dataset + cohort + platform + age.continuous + sex. For other models, the variable(s) in addition to the baseline variables are shown with the corresponding variance explained (*R*^2^) in Δage. Interaction terms with chronological age are modeled as a continuous variable (age.cont) or a categorical variable (age.groups). The latter uses previously defined decades. Model comparison is performed to assess whether the contribution of an interaction term is significant compared to a model without that term. The Chi-square test is used to test two models with corresponding *p*-value presented. The results of these analysis are shown for both the Horvath and Levine clock. *P*-values are corrected for the number of tests performed (3 clocks × 4 comparisons = 12)

**Fig. 3:**
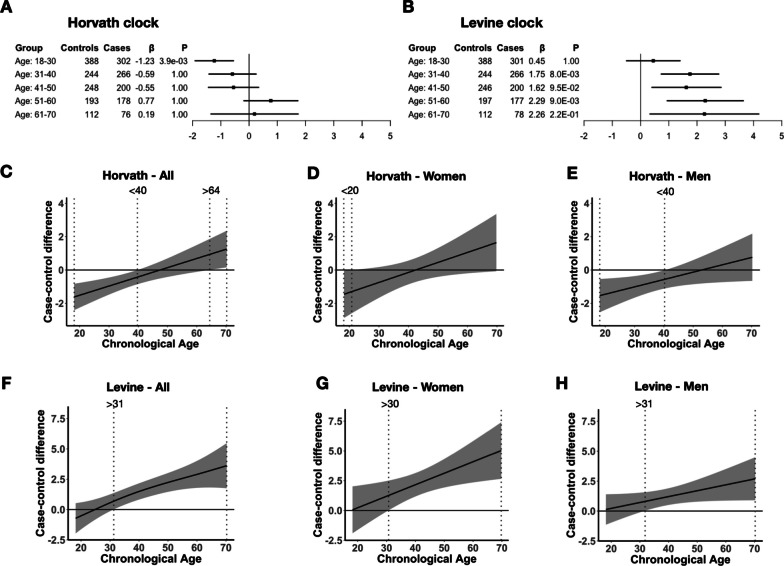
Differential DNAm aging in schizophrenia maps to specific age windows between sexes. **A**, **B** Shown are Δage differences between cases and controls across age groups for the Horvath (**A**) and Levine clock (**B**). For each age group, number of cases and controls, and meta-analytic effect size (*β*) and *p*-value (*P*) are presented. *P*-values are corrected for multiple testing (2 clocks × 5 groups = 10 tests). See Table S5 for more details on results and corresponding statistics. **C**–**H** Case–control Δage difference curves are visualized based on GAM predictions in the combined sample. Age intervals in which the 95% confidence interval of the difference estimate is different from zero are highlighted by dotted vertical lines. Identified age intervals for the Horvath and Levine clock are shown in (**C**–**E**) and (**F**–**H**), respectively. Results are shown for the full sample (left), women (middle), and men (right)

To further investigate age-specific effects, we modeled the interaction effect between disease status and chronological age on Δage using linear regression analyses and found a differential rate of aging between cases and controls (Additional file 2: Fig. S5). That is, the slope of Δage across chronological age is 0.05 and 0.06 years steeper in cases compared to controls for the Horvath (P_meta_ = 2.3E−03) and Levine clocks (P_meta_ = 7.1E−03), respectively, with no evidence of heterogeneity between cohorts (Additional file 2: Fig. S6 and Additional file 3: Table S8). As no significant effects were observed for the Hannum Δage, we decided to focus our downstream analysis on the phenotypic (Levine) age and intrinsic cellular (Horvath) age only. Next, we categorized chronological age into age groups by 10-year intervals and estimated differential aging between cases and controls within each group. We observe significant DNAm age deceleration in early adulthood (18–30 years) with patients estimated at − 1.23 years younger (P_meta_ = 3.9E−03) in intrinsic cellular age with no significant difference at later ages (Fig. 3A and Additional file 1: S7). In phenotypic age, SCZ patients displayed significant DNAm age acceleration from 30 years and older (Fig. 3B), with the most pronounced age acceleration between 50 and 60 years (2.29 years, P_meta_ = 9.0E−03). We again find no evidence of heterogeneity within age groups between cohorts (Additional file 2: Fig. S8 and Additional file 3: Table S9, 10).

To model a possible nonlinear relationship between DNAm aging and schizophrenia across the full range of chronological age, we implemented generalized additive models (GAMs) to estimate the trajectory of Δage and tested whether these trajectories were significantly different between cases and controls. We found significant nonlinear DNAm aging across chronological age for all three clocks (Additional file 2: Fig. S9, Additional file 4: Table S11). We, however, did not observe a significant interaction effect of case–control status with chronological age in the full sample, nor in men and women separately (Additional file 4: Table S12). Δage difference estimates between cases and controls, based on the GAMs, did identify specific age windows that suggest differences between men and women (Fig. 3C–H). Specifically, we found intrinsic age deceleration in men between age 18–40 (Fig. 3E) and more pronounced phenotypic age acceleration in women after age of 30 (Fig. 3G).

### DNAm aging affects SCZ above and beyond smoking and blood cell types

To investigate the effect of smoking and blood cell type composition, we use DNAm-based smoking and cell type estimations (see Methods) as a proxy to evaluate their contribution to DNAm aging in SCZ. While DNAm clocks, by design, will encapsulate such effects, quantifying the contributions of each factor increases interpretability and helps understand the factors contributing to the differential aging findings. We observe that blood cell type proportions explain significantly more variance in DNAm aging than DNAm smoking scores (Additional file 5: Supplementary Results S2.1). Inclusion of DNAm smoking score and blood cell proportions as covariates in our main models explains part, but not all of the observed disease effects (Additional file 1: Table S13). Using a penalized regression framework (Additional file 1: Table S14), we show that Levine Δage independently contributes to the variance in disease status in women older than 31 above and beyond smoking scores and blood cell type proportions (*P* = 5.5E−03) (Additional file 5: Supplementary Results S2.2 and Additional file 2: Fig. S10). A significant proportion of the Horvath Δage effect on disease status is reduced by adjusting for smoking (Additional file 1: Table S13-14). However, smoking is not associated with Horvath Δage in controls (Pearson *r* = 0.01, *P* = 0.95) nor in cases (Pearson *r* = − 0.08, *P* = 0.28) (Additional file 2: Fig. S11). As smoking covaries with SCZ disease status, it is difficult to distinguish these signals.

### Age deceleration by multi-tissue Horvath clock is not present in brain

We investigated DNAm aging in frontal cortex postmortem brain samples of 221 SCZ cases and 278 controls. The multi-tissue Horvath clock accurately predicts DNAm age in the brain as well (*r* = 0.94, *P* < 2.2e−16). We, however, find no difference in DNAm aging between cases and controls (*ß* = − 0.29, *P* = 0.46) and no evidence of age-dependent aging. More details are shown in Additional file 5: Supplementary Results (S2.3).

### Phenotypic age acceleration is associated with SCZ polygenic risk in women

**Fig. 4:**
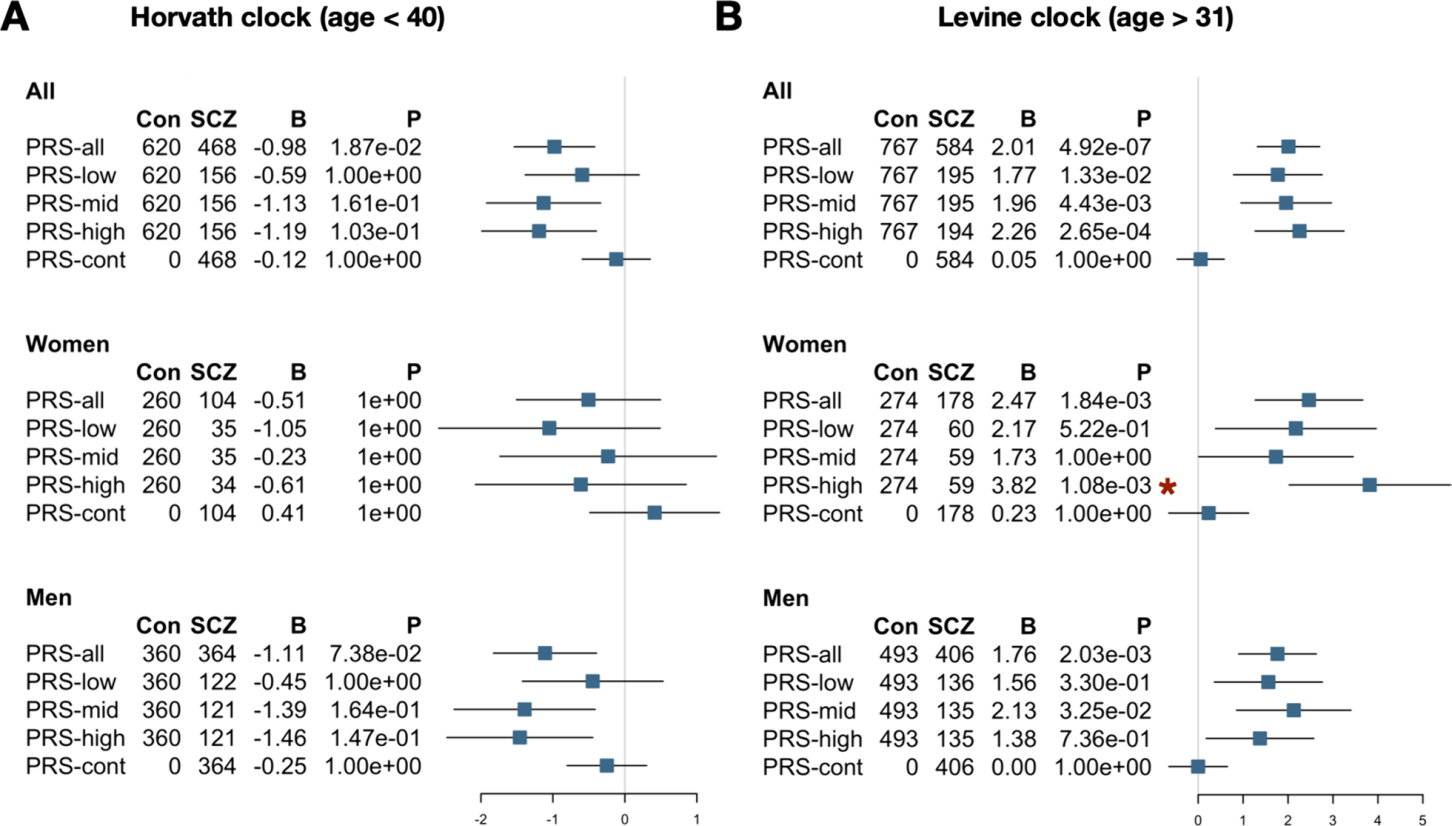
Phenotypic age acceleration associates with schizophrenia in women with high PRS. Forest plots showing associations statistics of Horvath Δage (**A**) and Levine Δage (**B**) with SCZ PRS stratified by sex. The analyses of Horvath Δage and Levine Δage were subsetted to individuals younger than 40 and older than 31 years, respectively. Analyses were furthermore stratified by sex. Case–control analyses were performed without PRS1 stratification (PRS-all), with cases in the first PRS tercile (PRS-low), cases in the second tercile (PRS-mid), cases in the third tercile (PRS-high), and using PRS1 as continuous variable in cases only. For each analysis, the number of controls and cases included, the regression coefficient, and the *P*-value are shown. *P*-values were adjusted for multiple testing (number of tests = 30). The red asterisk highlights analyses that are also significant after permutation (*P* < 0.05)

To further decipher the factors underlying the signal of differential aging in SCZ, we examined the role of SCZ polygenic risk (Additional file 2: Fig. S12, Fig. 4). Based on the identified age windows of differential aging between cases and controls, we performed polygenic risk association analyses with Horvath intrinsic cellular aging in individuals below the age of 40 and Levine phenotypic aging in individuals after the age of 31, and stratified our analyses by sex. As expected, PRS1 was significantly higher in cases compared to controls (odds ratio = 2.55, *P* = 1.9 × 10^106^) and explained almost 20% of the variance in disease status in the full sample (see Additional file 5: Supplementary Methods S1.6). We did not observe a significant association between intrinsic cellular aging and SCZ polygenic risk terciles or continuous PRS1 (Fig. 4A). We did find increased phenotypic age acceleration in cases with high SCZ genetic risk (Fig. 4B). Female cases in the highest PRS1 tercile are predicted to be + 3.82 years older in phenotypic age compared to matched controls (*P* = 1.1E−03), while women with SCZ with mid or low PRS did not show significantly different aging (Fig. 4B). By permutation of PRS1 terciles, we find that the effect in the highest PRS1 tercile is unlikely to occur by chance (*P* = 0.025). We do not observe such an association in men.

**Fig. 5:**
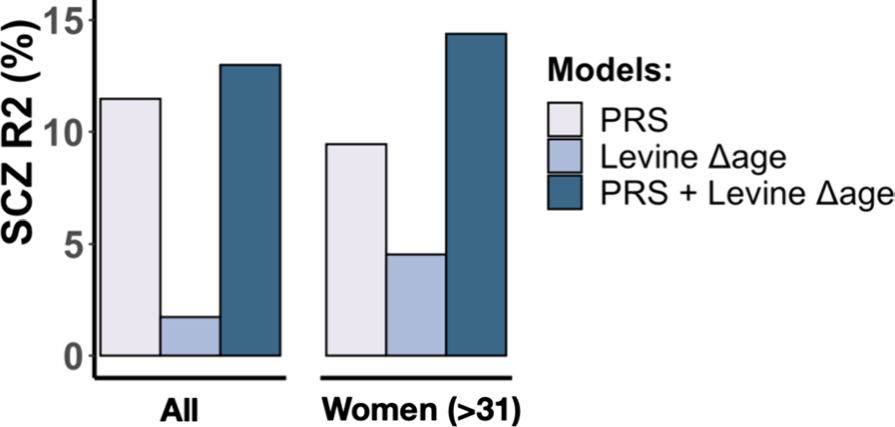
DNAm aging and polygenic risk contribute additively to schizophrenia. The variance explained in schizophrenia disease status (y-axis) by SCZ PRS1 and Levine Δage shown for all samples (left) and for women older than 31 years (right). The estimates shown are derived on top of the effect of technical variation, cohort, platform, and chronological age

Finally, we assessed how Levine Δage and SCZ PRS1 compare in predicting SCZ disease status in our sample. In the subsample with both polygenic risk and Levine DNAm age information available, PRS1 and Levine Δage explain 11.5% and 1.7% of the variance in disease status, respectively. Together, they explain 13.0%. In women in later adulthood, SCZ PRS1 and Levine Δage explain 9.4% and 4.5% independently and 14.4% jointly (Fig. 5).

## Discussion

We performed a large study of biological aging in schizophrenia using multiple epigenetic clocks based on whole blood DNA methylation data. Our study has several novelties. First, we performed a meta-analysis across four European cohorts as opposed to individual analyses per cohort, which, combined with multiple testing correction, is robust to cohort-specific artifacts in the data. Second, we performed detailed phenotypic analyses including explicit modeling of age- and sex-specific effects. We observe significant nonlinear patterns of age-specific DNAm aging in SCZ, a finding consistent across four European cohorts, that seems to have sex specificity as well. The most significant differential aging pattern that we observe is in cases ages 30 years and older, particularly in women, in which we detect advanced *phenotypic age acceleration*, as measured by the Levine clock. We also observe *intrinsic cellular age deceleration*, particularly in men, in SCZ during early adulthood in ages 40 and younger, as measured by the Horvath clock. Third, we integrated DNAm age with duration of illness and SCZ polygenic risk. We did not find an association between DNAm aging and duration of illness in patients. We did observe, however, that phenotypic age acceleration in women with a SCZ diagnosis is associated with a higher burden of SCZ polygenic risk. This high SCZ risk group has accelerated aging of + 3.82 years compared to age- and sex-matched controls. Phenotypic age and SCZ PRS furthermore contribute additively to SCZ and explain up to 14.4% of the variance in disease status. The latter suggests that combining genetic and epigenetic predictors can augment downstream prediction of outcomes in SCZ, similarly to what was recently shown for BMI [39].

Our results of significant phenotypic age acceleration in SCZ replicate a previous report on analysis of the Levine clock that was performed in a smaller but overlapping SCZ sample [35]. Similar to our finding of 1.53 years of phenotypic age acceleration in SCZ, they report a 1.4- to 1.9-year increase in Δage in SCZ cases compared to controls. Our meta-analysis showed that this effect is robust across four cohorts and has age and sex specificity, and associates with SCZ polygenic risk in women in later adulthood. The Levine estimator was constructed by predicting a surrogate measure of phenotypic age, which is a weighted average of 10 clinical markers, including chronological age, albumin, creatinine, glucose and C-reactive protein levels, alkaline phosphatase, and various blood cell-related measures [17]. By design, the Levine estimator is a composite biomarker that strongly predicts mortality, in particular that of age-related diseases, such as cardiovascular-related phenotypes. A 1-year increase in phenotypic age is associated with a 9% increased risk of all-cause mortality and a 10% and 20% increase of cardiovascular disease and diabetes mortality risk, respectively [17, 40]. The multiple year increase in phenotypic age in SCZ could thus imply an increased mortality in patients that is linked to cardiovascular disease, a previously well-established epidemiological observation [4–6]. A recent study, however, found that DNAm age acceleration only predicts mortality in SCZ cases without preexisting cancer using the Hannum clock [41]. They did not find such evidence using the Levine clock. The smaller sample size and predominantly male cohort, particularly given our finding of more pronounced age acceleration in women, may have reduced the predictive power of the study. The findings of this study, however, align with the observation that patients with SCZ, particularly women in later adulthood, are reported to be at increased mortality risk due to cardiovascular disease and diabetes [5, 42, 43]. Thus, changes in phenotypic age we observe in this group of SCZ patients may reflect a molecular signature related to this epidemiological observation. A more focused and larger study of DNAm aging in women with SCZ in later adulthood, preferably stratified by SCZ genetic risk, is, however, warranted to further understand the molecular mechanisms as well as the clinical utility of measured accelerated phenotypic age. Assuming that cardiovascular risk is modifiable in SCZ [44], phenotypic age could serve as a potential biomarker to identify at-risk individuals and in this way help with disease management and improvement of life expectancy.

In contrast to *age acceleration* in phenotypic age, we observe *age deceleration* in intrinsic cellular age (i.e., the Horvath DNAm age), which replicates results of previous studies [35, 36]. We show that men with SCZ in younger adulthood are particularly impacted and show the most significant deceleration, which was a robust finding across the four cohorts. Unlike the association findings in women, we did not observe clear patterns with genetic and phenotypic variables that could help to further decipher the signal. Unlike Wu et al. [36], we did not observe *age deceleration* in postmortem brain samples of the human cortex, indicating that the observed aging signal in SCZ may be blood-specific. However, our analysis in brain tissue was performed on a smaller sample but overlapping sample. Horvath DNAm aging has been shown to be associated with molecular processes of development and cell differentiation [15, 38], including blood-based DNAm aging in human (neuro)developmental phenotypes [45, 46]. One possible explanation is that patients diagnosed with SCZ in this age group show evidence of delayed or deficient development and that this is detectable in blood through the multi-tissue Horvath clock. Another explanation is that medication effects underlie biological age deceleration in SCZ, as clozapine use has been associated with Horvath intrinsic age deceleration in men [35]. More study is therefore needed to further dissect how blood-based *Horvath age deceleration* is associated with SCZ.

While we did observe aging effects with the Horvath and Levine clock, we did not find the same with the Hannum clock. The Hannum clock is less predictive of age acceleration effects on mortality risk than the Levine clock [17], which could explain the lack of findings in our analyses. The Hannum estimator furthermore cannot be used on first generation 27 K DNA methylation arrays which reduced the sample size of this study with 30% and may have impacted the statistical power of these specific analyses. This highlights the benefits of designing methods that are inclusive to all platforms, so all data, both old and new, can be leveraged.

A systematic review of aging biomarkers in SCZ found that only about a quarter of studies explored an interaction effect or statistically compared the regression slope between cases and controls [14]. Our findings of age- and sex-specific DNAm aging support their recommendations to specifically examine interaction effects with age and sex in aging studies but also more general in epigenetic studies of SCZ, such as epigenome-wide association studies. Results from our GAM analyses furthermore suggest that future research should aim to integrate nonlinear models to fully capture the complex relationship between DNAm aging and clinically relevant variables across the lifespan of patients. These models could help validate and further refine the most relevant age intervals in larger cohorts.

We did not observe an association between duration of illness and changes in DNAm age in SCZ. This lack of association is consistent across cohorts and suggests that the cumulative consequences of disease do not contribute to the observed acceleration in DNAm age. This is a surprising finding, as we hypothesized that disease-related effects could compound over time in individuals with a diagnosis of SCZ, thereby accelerating their biological age. However, it should be noted that illness duration and chronological age are highly correlated (rho = 0.84 in the full sample). As we adjusted for chronological age in our statistical models, we removed a significant proportion of the variation in duration of illness, which likely reduced statistical power to detect associations. This is a limitation of the cross-sectional design of the study and warrants further investigation using a longitudinal study design.

Another limitation of the cross-sectional design of the study is that dissecting cause-and-effect relationships between DNAm aging and SCZ remains challenging. As the published studies on SCZ DNAm aging that have so far been published in literature have been conducted on similar datasets, there is also a need for further replication and mechanistic investigation in new cohorts. Ideally, future studies should preferably use longitudinal prospective cohorts with genomic data and information on symptom recurrence and severity, comorbidities, and other phenotype-related variables. Such an approach can assess the clinical relevance of DNAm aging in SCZ above and beyond other known health risk factors and disease biomarkers, such as medication use and smoking. Our study, for example, did not include GrimAge, a recently trained DNAm mortality clock [47]. Unlike phenotypic age acceleration, GrimAge acceleration is largely driven by smoking effects. Thus, studies that investigate GrimAge in the context of SCZ should incorporate detailed information on smoking behavior. As this study consists of only European cohorts of primarily White study participants, future studies in other populations and racial–ethnic groups are needed to determine how generalizable our findings are. An urgent open question remains whether DNAm age signatures are modifiable with regard to clinical and lifestyle factors associated with SCZ. Improvement of existing methodology and/or development of new DNAm age biomarkers [48, 49] may in addition help to better study differential aging in SCZ and related disorders with increased mortality. Combining blood-based DNAm age with that of other aging profiles, such as MRI-based brain age [50], may further advance our understanding of aging and SCZ disease progression, including the increased mortality [51]. Finally, our findings support an integrative strategy with polygenic disease risk to improve clinical utilization.

Schizophrenia, like other mental illnesses, are associated with a wide-range of subsequent chronic physical conditions, including many age-related diseases [52]. While health and life expectancy of the general population continue to improve, the mortality disparity between patients with schizophrenia and those unaffected continues to increase [9, 10, 42, 53]. As the burden of age-related diseases continues to rise, early detection and subsequent opportunities for interventions before disabilities and comorbidities become established will be important [1, 2]. Molecular biomarkers of aging, such as DNAm clocks, are now emerging as candidate tools for screening and intervention. Taken together, this study strengthens the need for more research on DNA methylation aging in schizophrenia, a population vulnerable to age-related diseases and excess mortality.

## Material and methods

### Cohort and sample description

Details of samples included in this study can be found in Additional file 5: Supplementary Information. Briefly, unrelated patients with a diagnosis of SCZ and ancestry-matched non-psychiatric controls from four European cohorts were included: the Netherlands (*N* = 1116), Scotland (*N* = 847), Sweden (*N* = 96), and the UK (UK, *N* = 675). The Dutch DNAm cohort is a case–control sample with inpatients and outpatients recruited from different psychiatric hospitals and institutions across the Netherlands [54, 55]. All patients and controls were of Dutch descent, with at least three out of four grandparents of Dutch European ancestry. The controls were volunteers and free of any psychiatric history. The UK cohort is a case–control sample recruited from London and South England [56–58]. All subjects were included if both parents were of English, Irish, Welsh, or Scottish descent, with at least three out of four grandparents having the same origins. The Scottish cohort is a case–control sample of individuals that have self-identified as born in the British Isles (95% in Scotland) [56–58]. The Swedish cohort is a case–control sample of older study participants, i.e., 50–70 years. Cases were selected on the basis of a clinical diagnosis of SCZ using the Diagnostic and Statistical Manual for Mental Disorders (DSM-IV), Research Diagnostic Criteria (RDC), or the International Classification of Diseases 10 (ICD10). Controls were unaffected subjects without a history of any major psychiatric disorder. Whole blood DNAm data were available for a total of 2707 samples (1399 cases and 1308 controls; Additional file 1: Table S1).

### Genome-wide DNA methylation profiling and data processing

To quantify DNA methylation, DNA was extracted from whole blood and bisulfite converted for hybridization to the Illumina Infinium Human Methylation Beadchip. Samples were assayed with either the 27 K or 450 K beadchip, which contain 27,578 and 485,512 probes that interrogate CpG sites across the genome, respectively. For each platform, data processing pipelines were implemented, which includes background correction, color channel and probe-type correction, and normalization of the data, to minimize the effect of technical variation on the final beta values, as previously shown [59]. Samples with more than 5% of probes detected at *P* > 0.05 were excluded from further analyses (*n* = 13). Full details are described in Additional file 5: supplementary methods.

### DNAm-based estimation of biological age

To compute blood-based DNAm age estimates, processed beta values were used as input to the Hannum [16], Horvath [15], and Levine [17] DNAm clock. These DNAm age estimators use a set of CpGs that are selected via an optimization algorithm to collectively minimize the error associated with estimating chronological age (Additional file 5: Supplementary Information). Horvath DNAm age estimates were calculated using R scripts from the Horvath DNA Methylation Calculator (https://dnamage.genetics.ucla.edu). Hannum and Levine estimates were obtained by using the reported set of probes with corresponding regression weights. We define Δage by subtracting chronological age at the time of the blood draw from the predicted DNAm age.

### Statistical analyses

To investigate epigenetic aging differences in SCZ, we first removed samples with discrepant phenotypic sex/gender and predicted (biological) binary sex based on DNAm data (*n* = 9), as well as samples with missing chronological age data (*n* = 237), bipolar disorder diagnosis (*n* = 26), and duplicate samples (*n* = 126). For each epigenetic clock, we regressed Δage on technical principal components (PCs), using the first components that cumulatively explain > 90% of variation in intensity values of control probes, and added the residuals to mean(Δage) to generate a measure in the same units as Δage that is adjusted for technical variation (Δage-adjusted). We used the adjusted value for subsequent analyses and referred to it as Δage.

As association analyses of DNAm age between groups are sensitive to the distribution of chronological age, particularly at older ages, any case older than the oldest control was excluded from each cohort (*n* = 5 for NLD, 16 for SCT, 4 for SWD, and 1 for UK). Chronological age was furthermore included as a covariate in all analyses, as recommended [60]. To minimize the effect of outlying samples, we excluded samples > 3SD from mean Δage across cohorts (ranging from *n* = 13–16 for the three clocks). These are samples for which DNAm age diverged substantially from chronological age, which are likely artifacts.

For each clock and each cohort, we implemented a multivariable regression model predicting Δage as a function of schizophrenia status, sex, and age. For the Dutch cohort, batch and array platform were also included as covariates, as this cohort consists of multiple datasets from both the 27 K and 450 K platform. For each clock, regression coefficients with corresponding standard errors for each of the four cohorts were then supplied to the rma() function of the metafor package [61] in R to fit a meta-analytic fixed-effect model with inverse variance weights and obtain an overall effect size and test statistic.

To quantify the significance of age- and sex-specific effects, we determined the contribution of interaction effects on top of the main disease effect. We first combined all cohorts to maintain necessary sample sizes across age and sex groups. Age groups were defined by grouping samples by decades with ages 18 and 19 included in the first decade (18–30, 31–40, etc.). To quantify the gain in variance explained in Δage, models with the interaction term were compared to a baseline model without the interaction term. For each analysis, statistical significance was determined using Bonferroni correction, i.e., *P* < 0.05/number of tests.

GAMs were used to investigate nonlinear associations between DNAm aging and SCZ across chronological age. GAMs can identify trajectories in both longitudinal [62] and cross-sectional data [63] and identify difference between groups [64, 65]. We modeled Δage of each clock as a (potentially) nonlinear function of chronological age and tested for significant interaction effects of chronological age with case–control status. Difference plots were used to visualize differential aging between cases and controls within specific chronological age windows. Analyses were performed in the combined sample, and in men and women separately, using R_v4.0.1 using the packages mgcv_1.8.33 [64] and itsadug_2.4 [66].

### SCZ polygenic risk quantification

Polygenic risk scores (PRS) were obtained from analyses of the SCZ GWAS conducted by Psychiatric Genomics Consortium (PGC) [67]. Using a leave-one-out approach, weights were generated in a training dataset based on all samples minus the target cohort in which the PRS were calculated. For each individual, weighted single-nucleotide polymorphisms (SNPs) were summed to a genetic risk score that represents a quantitative and normally distributed measure of SNP-based SCZ genetic risk. To reduce between-cohort variation and maximize statistical power, we used a previously developed analytical strategy that uses principal component analysis (PCA) to concentrate disease risk across PRSs of ten GWAS *p*-value thresholds into the first principal component (PRS1) [68] (Additional file 5: Supplementary Information). PRS1 explains 70.7% of the variance in risk scores and 19.9% of the variance in SCZ status, which is more than any of the original p-value thresholds (4.9–17.4%). The other PCs had no explanatory value in disease status (mean *R*^2^ = 0.0%), which means that PRS1 captures the majority of SNP-based SCZ polygenic risk. PRS1 was generated for 1933 individuals, 853 cases and 1080 controls, and modeled as both a quantitative and categorical variable to predict Δage.

### Defining age at onset and illness duration

Age at onset is defined as the earliest reported age of psychotic symptoms or by the Operational Criteria Checklist (OPCRIT), depending on the cohort. These data are available for a subset of cases (*N* = 710) across the Dutch, Scottish, and UK cohorts. Illness duration is defined as the time between age at onset and blood collection. A more detailed description of each cohort’s definition is available in Additional file 5: Supplementary Information.

### DNA methylation-based smoking scores and blood cell type proportions

For samples assayed on the 450 K platform, smoking scores and blood cell type proportions were estimated from the data (see Additional file 5: Supplementary Methods) and used as a proxy to further decompose and understand differential aging effects in a subsample of the combined sample.

### DNAm age in postmortem brain case–control samples

DNAm age estimates were generated for case–control postmortem brain frontal cortex samples (*N* = 499) using publicly available data across four datasets (Additional file 1: Table S3 and Additional file 5: Supplementary Results S2.3).

### Estimating the contribution of differential aging in schizophrenia

Using a multivariable logistic regression model for disease status, we fitted batch, cohort, DNAm smoking score, DNAm blood cell type proportions, and Δage as explanatory variables. We first performed a variable reduction step to select the most contributing variables to disease status by use of a regularized logistic regression using the glmnet() function in R (“glmnet” package, v2.13) [69]. Alpha was set to “1” (Lasso) and the lambda parameter estimated at the optimal value that minimizes the cross-validation prediction error rate using the cv.glmnet() function. For each selected variable, we then report the variance explained in SCZ status (glm, family = “binomial”) for both the individual variable and adjusted for all other selected variables using the NagelkerkeR2() function in the “fmsb” package (v 0.6.3). The significance of each variable to their contribution was computed by comparing the model with and without the variable of interest using the likelihood ratio test of the anova() function.

### Supplementary Information

**Additional file 1**. Supplementary tables 1–3, 7, 13–14.

**Additional file 2**. Supplementary figures 1–12.

**Additional file 3**. Supplementary tables 5–6, 8–10: meta-analysis model statistics.

**Additional file 4**. Supplementary tables 11–12: generalized additive model statistics.

**Additional file 5**. Supplementary information - Methods and Results.

**Additional file 6**. Supplementary table 4: individual sample information and DNAm age estimates.

## Data Availability

The datasets used are available on the NCBI Gene Expression Omnibus (GEO) data repository or through the principal investigator of each cohort. See Additional file 1: Table S2 and S3 for an overview and corresponding accession series numbers. See Additional file 6: Table S4 for sample information, including individual DNAm age estimates.
