# Supplementary material for: Meta-analysis of epigenetic aging in schizophrenia reveals multifaceted relationships with age, sex, illness duration, and polygenic risk
Source: Clin Epigenetics. 2024 Apr 8;16:53. doi: 10.1186/s13148-024-01660-8 (PMC11003125; doi:10.1186/s13148-024-01660-8)
Supplement: Supplementary file 1 — Additional file 1. Supplementary tables 1–3, 7, 13–14. [file 13148_2024_1660_MOESM1_ESM.pdf]

	NLD	SCT	SWD	UK	Total
**Samples (N)**	926	665	69	636	2.296
**Cases**	461	260	37	332	1.090
**Controls**	465	405	32	304	1.206
**Age (years)**	35.8 (13.2)	44.6 (12.9)	59.7 (5.94)	40.4 (15.0)	40.3 (14.4)
**Cases**	35.5 (13.3)	44.2 (14.1)	59.6 (6.30)	43.7 (14.6)	40.9 (14.8)
**Controls**	36.1 (13.2)	44.9 (12.2)	59.9 (5.58)	36.8 (14.7)	39.8 (14.1)
**Females**	309 (33.4%)	185 (27.8%)	39 (56.5%)	259 (40.7%)	792 (34.5%)
**Cases**	122 (26.5%)	83 (31.9%)	20 (54.1%)	90 (27.1%)	315 (28.9%)
**Controls**	187 (40.2%)	102 (25.2%)	19 (59.4%)	169 (55.6%)	477 (39.6%)
**Hannum DNAm age**	37.9 (15.4)	46.0 (14.1)	61.7 (8.37)	41.6 (15.7)	43.1 (15.6)
**Hannum Δage**	0.0 (4.7)	1.4 (4.2)	2.0 (4.4)	1.2 (4.7)	1.0 (4.6)
**Horvath DNAm age**	37.0 (14.0)	47.6 (13.5)	62.0 (7.7)	41.2 (14.5)	42.0 (15.0)
**Horvath Δage**	1.2 (4.8)	3.0 (4.7)	3.2 (4.5)	0.9 (5.7)	1.7 (5.12)
**Levine DNAm age**	27.5 (15.1)	37.6 (15.4)	52.9 (8.1)	32.6 (16.5)	32.6 (16.4)
**Levine Δage**	-8.33 (6.7)	-7.0 (6.2)	-6.8 (5.4)	-7.8 (6.6)	-7.7 (6.5)

Table S1. Sample characteristics and DNA methylation age estimates across cohorts. Sample characteristic and mean values of chronological age and DNAm age estimates of each clock are presented for each cohort after quality control. Δage is defined by subtracting chronological age from DNAm age. Standard deviations are in parentheses unless otherwise defined. NLD = the Netherlands, SCT = Scotland, SWD = Sweden, UK = United Kingdom.

Dataset	PI/Contact	Ancestry	Platform	Data type used	Total	Cases	Controls	Age (sd)
GSE41037	RA Ophoff	Dutch	27K	IDAT files	624	337	287	33.3 (12.1)
GSE41169	RA Ophoff	Dutch	450K	IDAT files	96	62	34	31.1 (10.2)
TBD	RA Ophoff	Dutch	450K	IDAT files	324	160	164	34.4 (11.4)
TBD	RA Ophoff	Dutch	450K	IDAT files	72	36	36	59.2 (5.7)
TBD	PF Sullivan	Swedish	450K	IDAT files	96	48	48	59.8 (5.9)
GSE80417	A McQuillin	Scottish	450K	(un)methylated intensities	847	414	433	44.6 (12.9)
GSE84727	D St. Clair	UK	450K	(un)methylated intensities	675	353	322	40.4 (15.0)
Total		EUR	27/450K	Mixed	2.734	1.410	1.324	40.4 (28.1)

Table S2. Overview of datasets included in study. Multiple datasets of whole blood DNAm data across four European cohorts were included in the study. Shown above are some sample characteristics and accompanying GEO accession numbers for each dataset before quality control. PI = Principal Investigator, UK = United Kingdom, EUR = European.

Dataset	Ancestry	Platform	Tissue	Data type	Total	Cases	Controls	Age (sd)
GSE74193	AA/EUR	450K	DLPFC	IDAT files	503	224	279	46.9 (15.4)
GSE61107	-	450K	Frontal cortex	IDAT files	48	24	24	61.7 (19.2)
GSE61380	-	450K	Frontal cortex	(un)methylated intensities	33	18	15	44.0 (15.7)
GSE61431	-	450K	Frontal cortex	(un)methylated intensities	43	20	23	61.8 (17.5)
Total	Mixed / unknown	450K	Frontal cortex	Mixed	627	286	341	

Table S3. Overview of datasets included in brain analysis. Multiple datasets of postmortem brain DNAm data were included in in the analysis. Shown above are some sample characteristics and accompanying GEO accession numbers for each dataset before quality control. AA = African American, EUR = European, DLPFC = Dorsolateral prefrontal cortex.

lm(formula = Δage ~ Dataset + Cohort + Platform + Age.continuous + Status*Age.groups*Sex)

	Horvath Δage				Levine Δage			
**Model variables**	**Df**	**Sum Sq**	**Mean Sq**	**P-value**	**Df**	**Sum Sq**	**Mean Sq**	**P-value**
Dataset	6	1795	299,2	2,0E-15	6	679	113,2	6,7E-03
Cohort	-	-	-	-	-	-	-	-
Platform	-	-	-	-	-	-	-	-
Age.continuous	1	116	115,8	2,1E-02	1	1054	27,7	1,5E-07
Sex	1	46	45,7	1,5E-01	1	412	412,2	1,0E-03
Age.group	4	645	161,3	6,1E-06	4	512	128,0	9,3E-03
Status	1	288	288,0	2,8E-04	1	916	915,7	9,8E-07
Age.group:Status	4	227	56,7	3,4E-02	4	491	122,7	1,2E-02
Status:Sex	1	12	11,9	4,6E-01	1	51	50,9	2,5E-01
Age.group:Sex	4	329	82,1	4,5E-03	4	694	173,5	1,1E-03
Age.group:Sex:Status	4	66	16,3	5,5E-01	4	273	68,3	1,3E-01
Residuals	2135	46331	21,7	-	2137	81187	38,0	-

Table S7. Results three-way interaction model of age, sex, and status on Δage. Shown are the contributions of each variable in the three-way interaction model presented by an analysis of variance table. The full model is displayed in the top row. Age.groups are defined by decades. Cohort and Platform are collinear with Dataset and thus do not have output. Df = degrees of freedom; Sum Sq; sum of squares; Mean Sq; mean of squares; P-value corresponds to the F-test in the anova() function.

Model variables	Model comparison	Horvath Δage Δage R^2	Horvath Δage P-value	Levine Δage Δage R^2	Levine Δage P-value
Model x: baseline		3,9%	-	3,1%	-
Model y: baseline (+ smoking)	-	4,9%	-	5,3%	-
Model z: baseline (+ cell types)	-	8,2%	-	22,1%	-
Model 0: baseline (+ smoking/cell)	-	9,4%	-	22,8%	-
Model 1: + status	Model 0 vs 1	9,3%	0,86	22,8%	0,26
Model 2: + status*age.continuous	Model 1 vs 2	9,4%	0,10	23,2%	1,3E-03
Model 3: + status*age.groups	Model 1 vs 3	10,6%	2,1E-04	23,2%	0,05
Model 4: + status*age.groups*sex	Model 3 vs 4	10,8%	0,15	23,6%	0,05

Table S13. Age- and sex-specific effects of DNAm aging in schizophrenia adjusted for smoking and cell type estimastes. Shown are the contributions of interaction effects between disease status and age and sex on Δage when adjusted for DNAm smoking scores (baseline model y) and blood cell type proportions (baseline model z). The full baseline model is defined as Δage ~ dataset + cohort + age.continuous + sex + DNAm smoking score + DNAm blood cell type proportions. For other models, the variable(s) in addition to the full baseline variables are shown with the corresponding variance explained (R^2) in Δage. Interaction terms with chronological age are modeled as a continuous variable (age.continuous) or a categorical variable (age.groups). The latter uses previously defined decades. Model comparison is performed to assess if the contribution of an interaction term is significant compared to a model without that term. The chi-square test is used to test two models with corresponding p-value presented. The results of these analysis are shown for both the Horvath and Levine clock. These analyses included only 450K samples for which smoking scores and cell type estimates can be computed (N=1,621, 867 controls and 754 cases).

Women: >31 years (case = 190, control = 201)	Variable R2	Variable P	Variable R2 adjusted	P adjusted
Model - all selected variables	23.0%	5.9E-11	-	-
**Levine Δage**	**6.4%**	**1.2E-05**	**2,2%**	**5.5E-03**
Batch/Cohort	0.9 %	5.9E-01	2.1%	1.8E-01
Smoking	10.0%	3.6E-08	5.4%	1.5E-05
CD8.naive	0.05%	6.9E-01	0.6%	1.4E-01
CD4.naive	3.1%	2.6E-03	0.3%	2.8E-01
CD8T	7.1%	3.9E-06	0.7%	1.3E-01
NK	6.4%	5.8E-05	0.0%	0.9E-01
Granulocytes	7.7%	1.6E-06	0.5	1.7E-01

Men: <40 years (case = 302, control = 274)	Variable R2	Variable P	Variable R2 adjusted	P adjusted
Model - all selected variables	44.1%	1.0E-41	-	-
**Horvath Δage**	**1.45%**	**1.2E-02**	**0.2%**	**2.8E-01**
Batch/Cohort	3.5%	1.3E-02	1.6%	1.9E-02
Age	0.0%	7.5E-01	0.0%	7.1E-01
Smoking	30.1%	5.9E-35	16.8%	2.2E-23
CD8T	3.3%	1.6E-04	0.5%	8.8E-02
Granulocytes	5.2%	1.7E-06	1.3%	4.1E-03
CD8pCD28nCD45RAn	0.8%	5.7E-02	0.4%	1.0E-01
PlasmaBlast	4.0%	9.7E-01	1.6%	1.4E-03
NK	14.4%	4.7E-16	0.1%	3.3E-01
CD8.naive	0.2%	3.3E-01	0.2%	2.5E-01
CD4.naive	7.9%	2.8E-09	0.8%	2.7E-02

Table S14. DNAm aging significantly contributes to schizophrenia independent of smoking and cell types. Shown are variables that significantly explain variance in SCZ disease status, selected by a penalized logistic regression analysis. The top and bottom table present results for women >31 years and men <40 years, respectively. Only samples assayed on the 450K platform were included as DNAm-based smoking scores and cell type proportions could be computed and included in the analysis. The top row of each table shows the proportion of variance explained in disease status (R^2) for all selected variables combined and the significance of a logistic regression model (glm, family="binomial") with each variable included compared to the null model of no variance explained. We also show the proportion of variance explained by each variable individually (Variable R2) and by each variable adjusted for all other selected variables (Variable R2 adjusted). The significance of Variable R2 adjusted is computed by comparing the model with all variables to a model with the variable of interest removed using the anova(test = "LRT") function. The result of this test is shown in the "P adjusted" column.
