# Supplementary material for: Meta-analysis of epigenetic aging in schizophrenia reveals multifaceted relationships with age, sex, illness duration, and polygenic risk
Source: Clin Epigenetics. 2024 Apr 8;16:53. doi: 10.1186/s13148-024-01660-8 (PMC11003125; doi:10.1186/s13148-024-01660-8)
Supplement: Supplementary file 2 — Additional file 2. Supplementary figures 1–12. [file 13148_2024_1660_MOESM2_ESM.pdf]

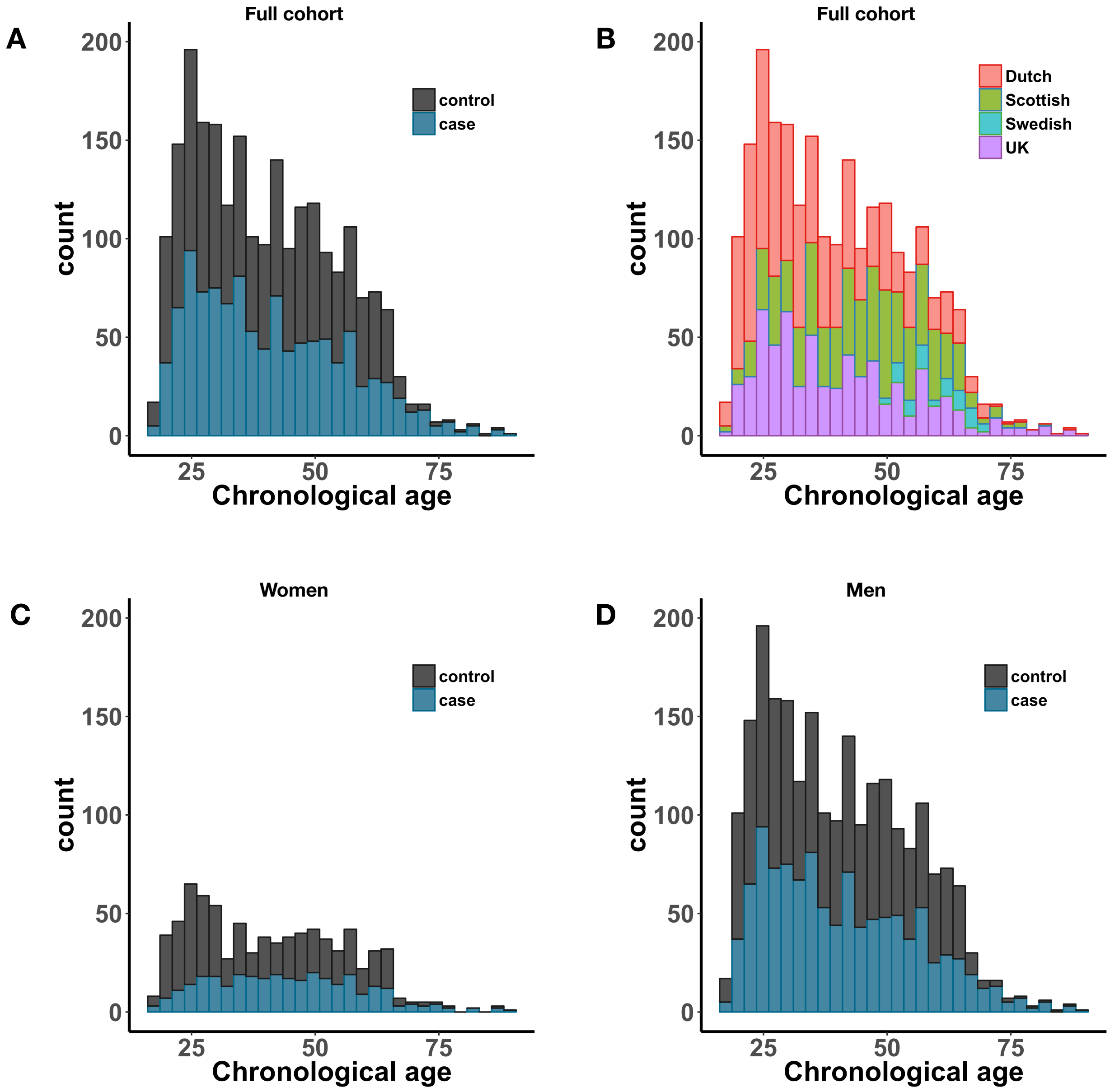

Figure S1. Sample distribution across chronological age. (A) Full sample colored by disease status. (B) Full sample colored by cohort. Women (C) and men (D) colored by disease status. UK = United Kingdom.

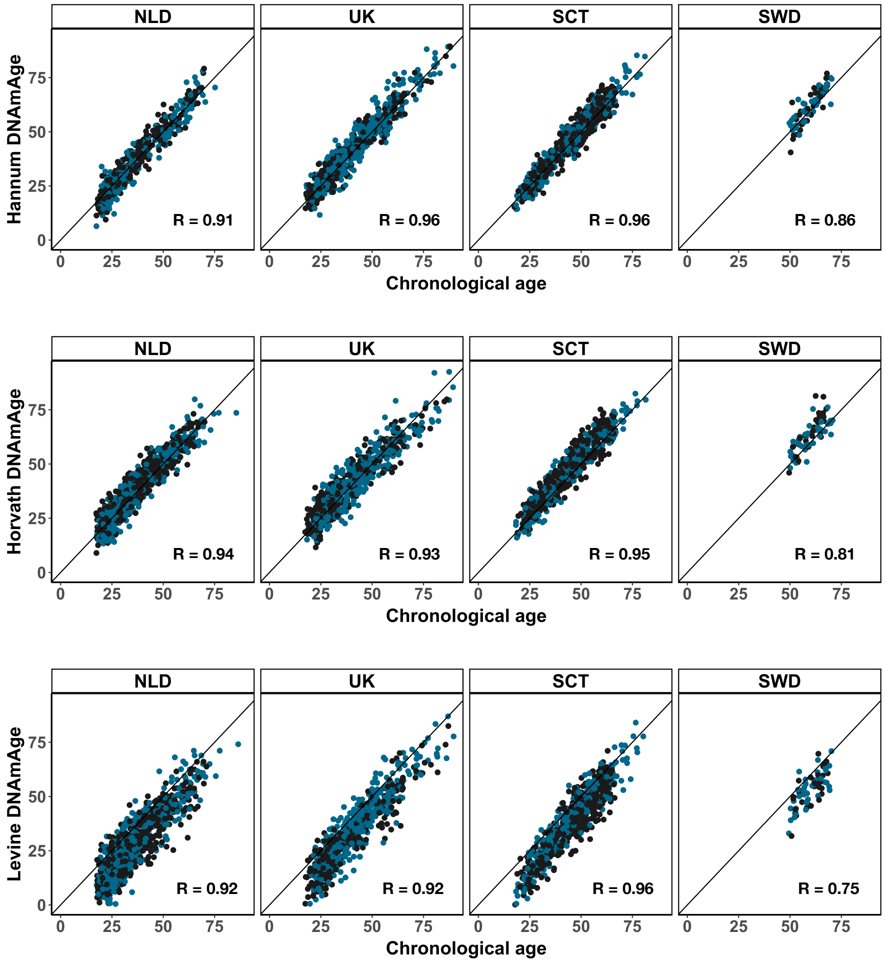

Figure S2. Correlations between DNAm age and chronological age by cohort. Shown are correlations for the Hannum (top), Horvath (middle), and Levine clock (bottom) across cohorts. NLD = Netherlands, UK = United Kingdom, SCT = Scotland, SWD = Sweden.

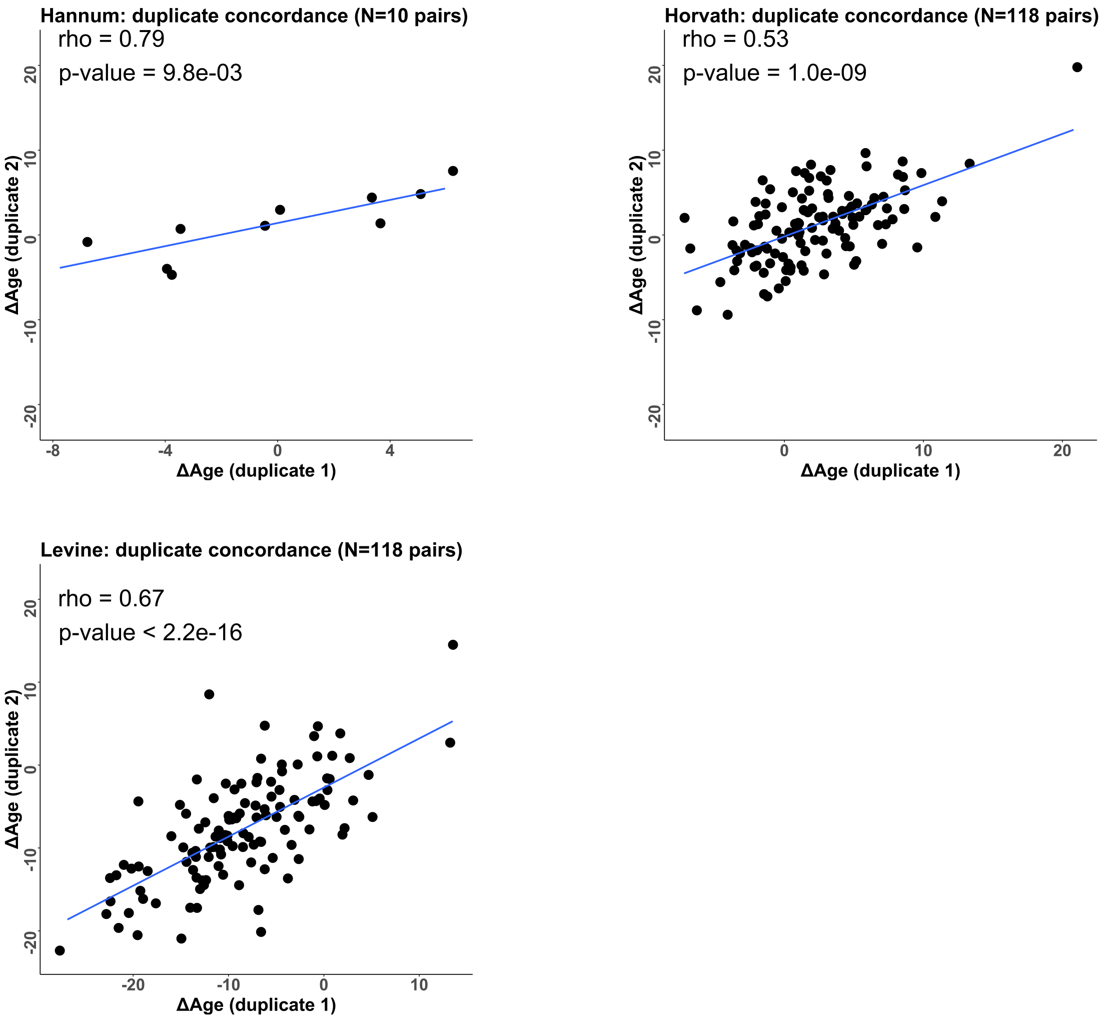

Figure S3. Sample duplicate pair concordance for DNAm age estimates of the Horvath and Levine clock. Using Δage across 118 duplicate pairs, the concordance between pairs is shown for the Horvath (top-left) and Levine (top-right) plot. For the Hannum clock (bottom), only duplicates with both samples on the 450K array (N=10) could be used.

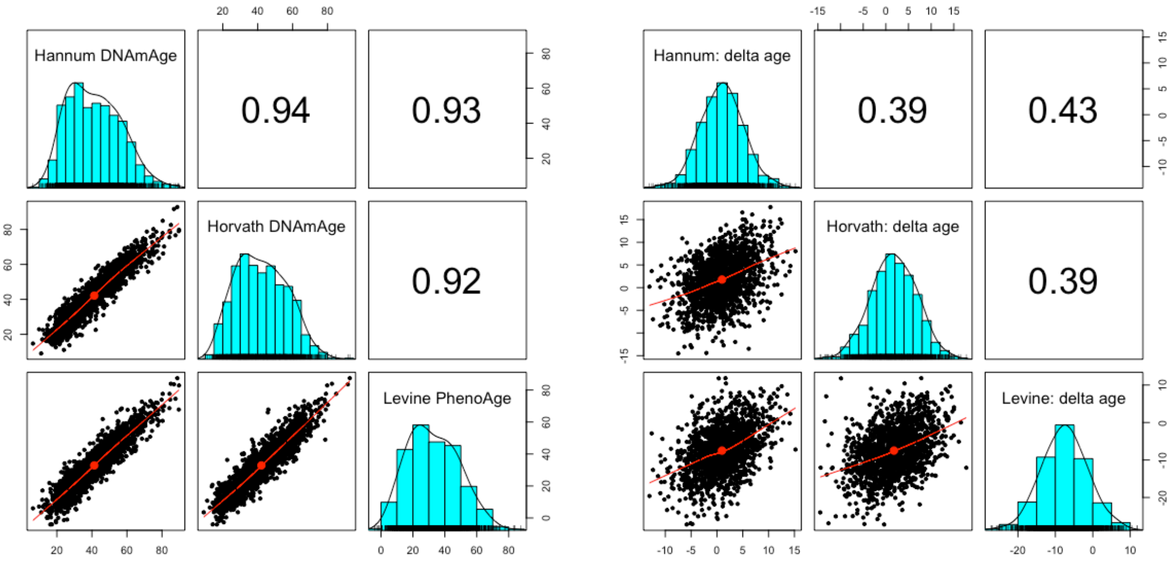

Figure S4. Correlation structure across clocks highlights that they capture both shared and distinct aspects of aging. Shown are pair-wise scatter plots below the diagonal, histograms on the diagonal, and the Pearson correlation above the diagonal for DNAm age (left) and Δage (right) across the three clocks.

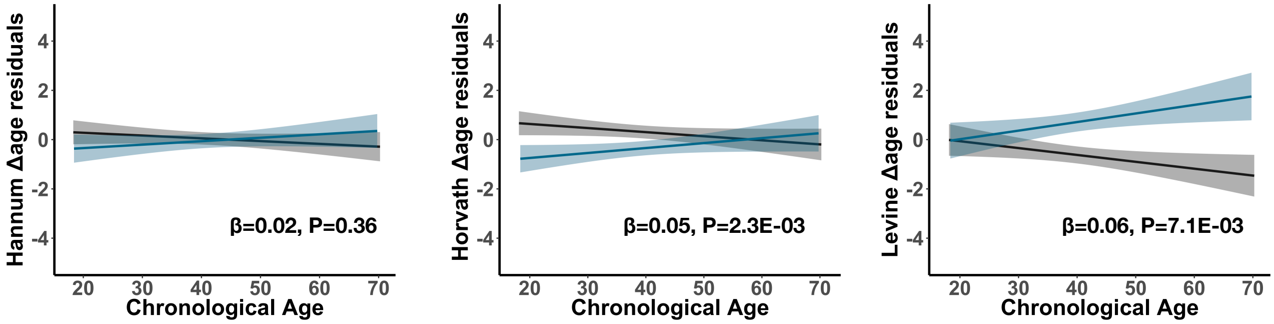

Figure S5. Differential DNAm aging across cohorts. Shown are results for modeling the interaction between disease status and chronological to estimate Δage differences between cases and controls conditional on age. For each estimator - Hannum (left), Horvath (middle), Levine (right) - Δage is visualized across chronological age with a regression line fitted separately for cases and controls and the meta-analytic interaction effect and p-value shown. β represents the change in Δage in cases per year of chronological age compared to controls. P-values are adjusted for multiple testing across clocks (n=3).

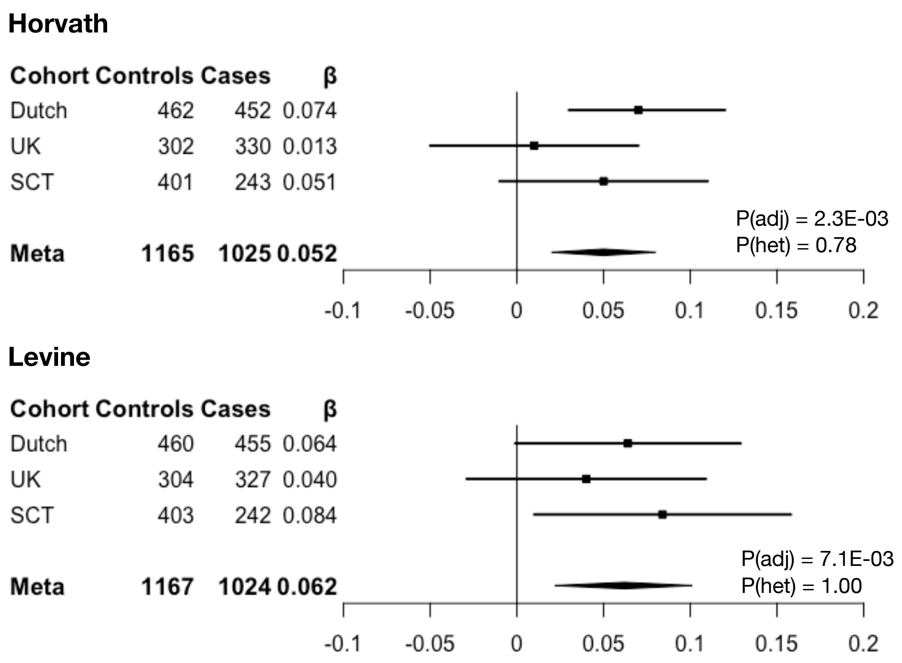

Figure S6. Differential DNAm aging across cohorts. Shown are results for modeling the interaction between disease status and chronological to estimate Δage differences between cases and controls conditional on age. For each estimator - Hannum (top), Horvath (middle), Levine (bottom) - number of cases and controls, and meta-analytic effect size (β) and adjusted p-value (Padj) are presented. See Table S4 for more details on results and corresponding statistics. The Swedish cohort was excluded from this analysis as it has only a limited spread in age (i.e. 50-70 years). Dutch = Netherlands, UK = United Kingdom, SCT = Scotland.

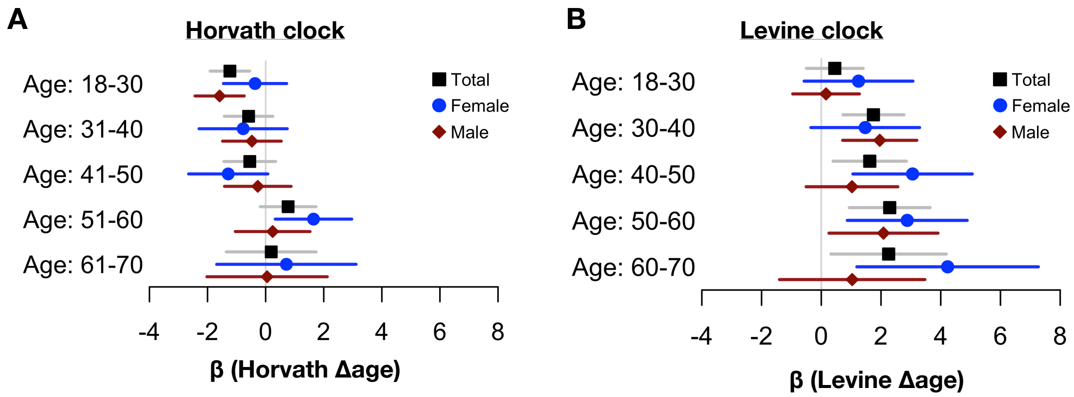

Figure S7. Sex-stratified differential aging by age groups in schizophrenia. Shown are Δage differences between cases and controls across age groups stratified by sex for the Horvath (A) and Levine clock (B). Results for women and men are presented in blue and red, respectively. The effects in the total sample are displayed in black.

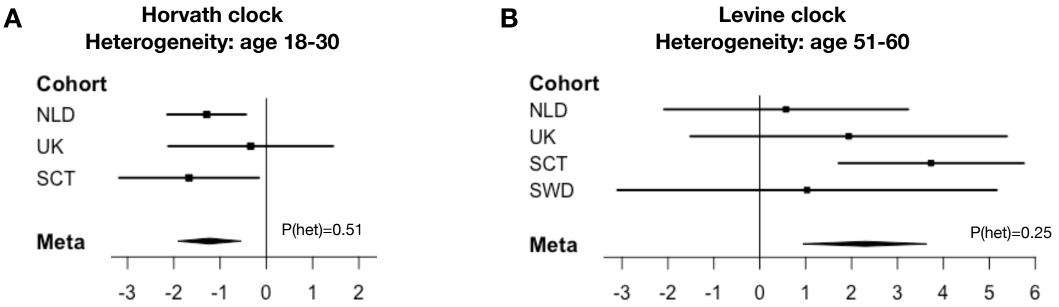

Figure S8. Aging effects within age groups are similar across cohorts. Shown are results for Δage differences between cases and controls in specific age groups for the Horvath (left) and Levine (right) clock. Each forest plots show a significant meta-analytic effect size and the p-value of Cochran's heterogeneity test (Phet). See Table S7 and S8 for more details on results and corresponding statistics. The Swedish cohort was excluded from the left plot as it has only a limited spread in age (i.e. 50-70 years). NLD = Netherlands, UK = United Kingdom, SCT = Scotland.

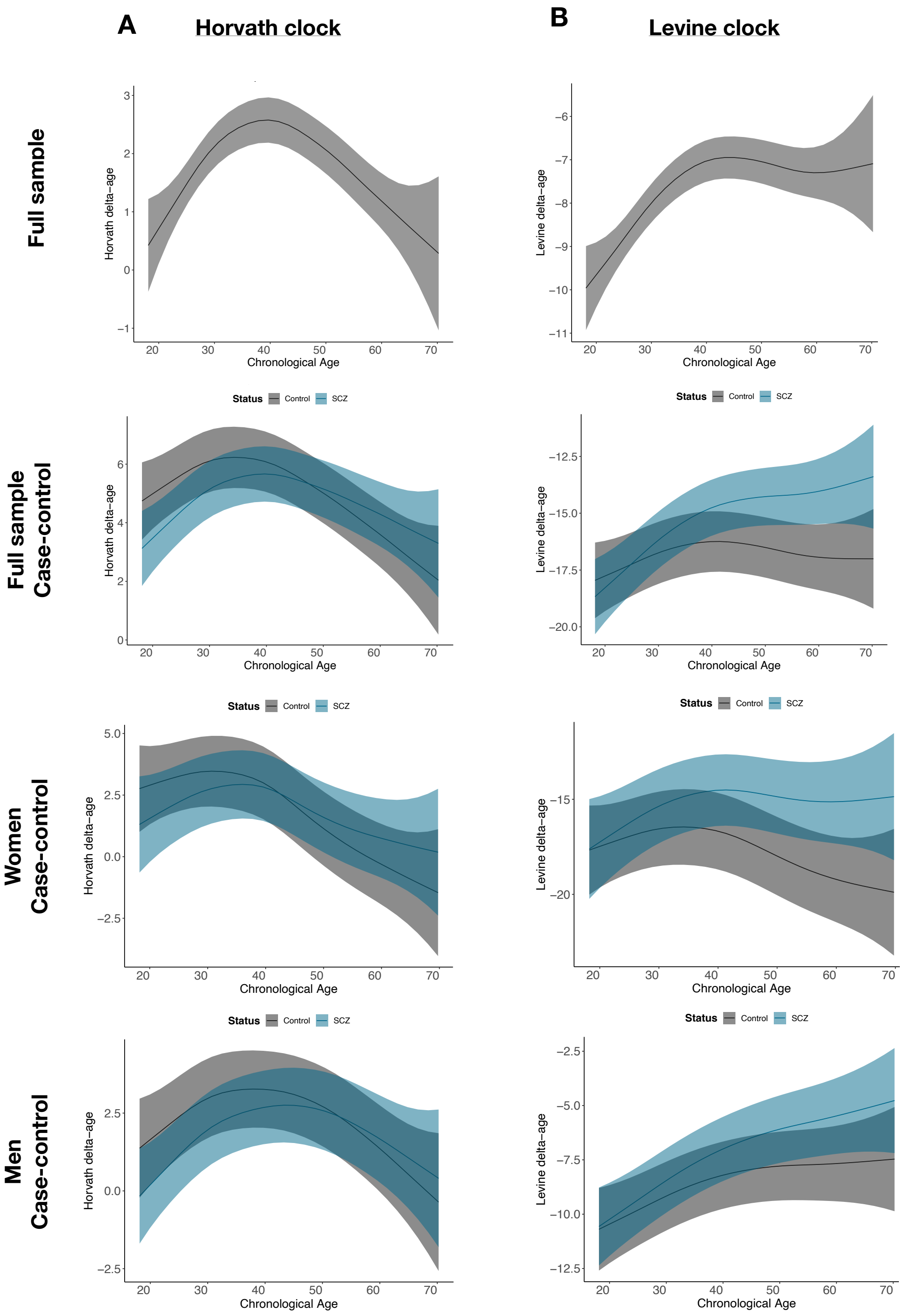

Figure S9. Generalized additive model trajectories of DNAm aging in schizophrenia. Shown are estimates of the GAM models in the full sample (cases and control combined), in the full samples for cases and controls separate, and for women and men for the Horvath clock (left) and Levine (right). The mean estimate with 95% confidence intervals are shown. SCZ is blue, controls are in dark grey.

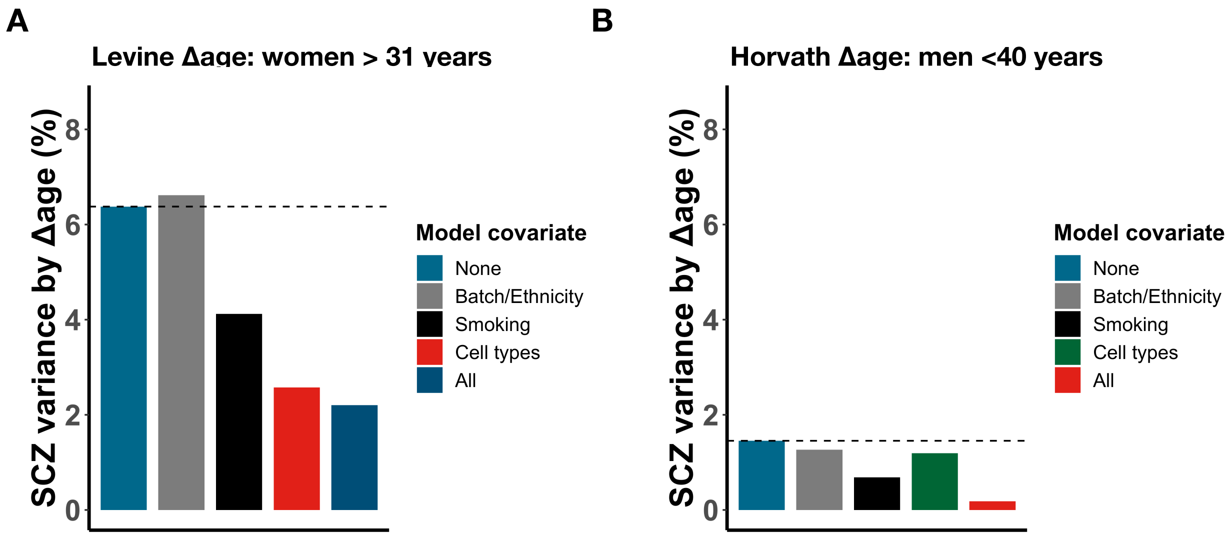

Figure S10. Smoking and blood cell type composition contribute in part to DNAm aging. Presented are the results of a sensitivity analysis of DNAm-based estimated smoking score and blood cell type proportions in the 450K subsample of the cohort. (A/B) The proportion of schizophrenia variance explained by Δage after adjustment of various variables that are significantly associated with disease status. The "All" model presents the variance explained by Δage independent from all other variables. Results are shown for Levine Δage women > 31 years, 190 cases and 201 controls and Horvath Δage men < 40 years, 302 cases and 274 controls separately.

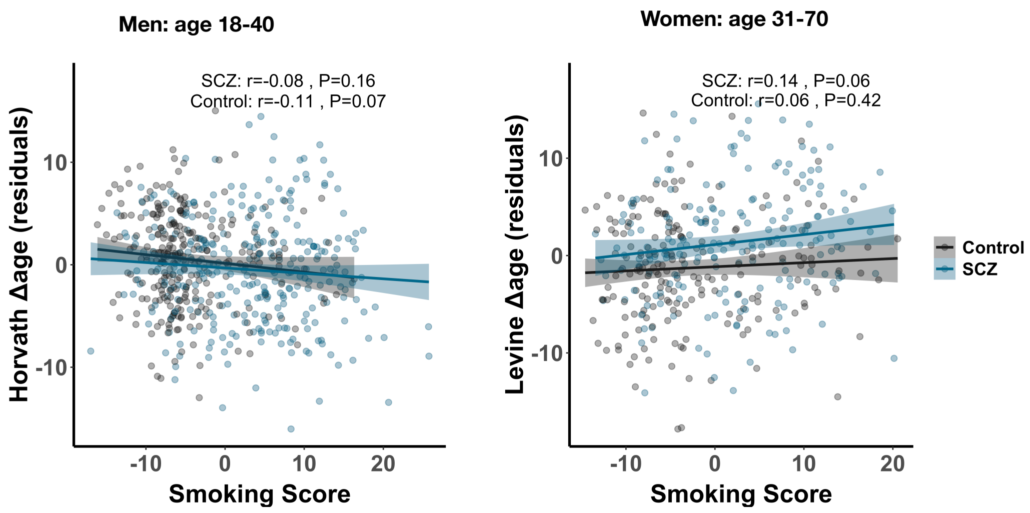

Figure S11. Smoking has only minimal effects on DNAm aging. Shown is the relationship between Δage and DNAm-based smoking score for men (left plot; age 18-40, case = 302, control = 274) and women (right plot; age 31-70, case=190, control=201). DNAm Δage was first regressed on batch, cohort and chronological age. The Pearson correlations and corresponding p-values are shown on top of each plot. Cases and controls are presented in blue and black, respectively.

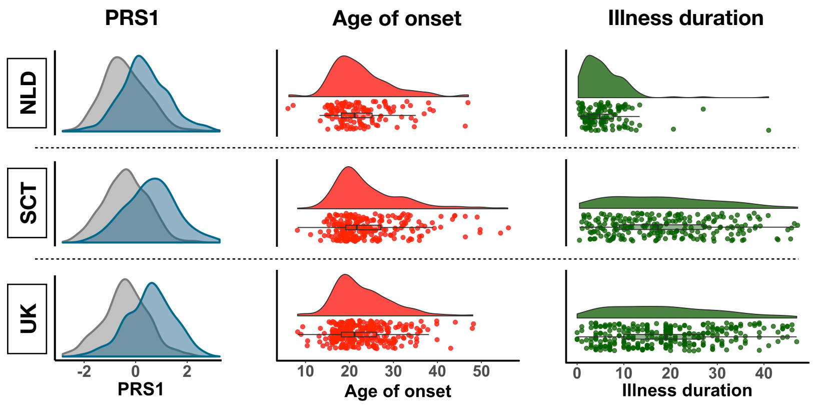

Figure S12. Variable distribution of PRS, age of presentation, and illness duration. The distribution of SCZ PRS1 score (left; cases in blue), age of presentation (middle), and illness duration (right) for each of the three cohorts with available information. NLD = Netherlands, UK = United Kingdom, SCT = Scotland.
