# Supplementary material for: Meta-analysis of epigenetic aging in schizophrenia reveals multifaceted relationships with age, sex, illness duration, and polygenic risk
Source: Clin Epigenetics. 2024 Apr 8;16:53. doi: 10.1186/s13148-024-01660-8 (PMC11003125; doi:10.1186/s13148-024-01660-8)
Supplement: Supplementary file 4 — Additional file 4. Supplementary tables 11–12: generalized additive model statistics. [file 13148_2024_1660_MOESM4_ESM.docx]

**Table S11. GAM estimates of Δage trajectories in the full sample across chronological.** Shown are the statistical output of the GAM for Horvath (left) and Levine (right) Δage in the full sample with only age (top table) and with the interaction of age and disease status (bottom table). The trajectory of Δage was modeled using a single smooth term of chronological age (in years) as predictors and a combined smooth term of chronological age and disease status. Shown are the estimate and standard error (SE) of the intercept and the fitted smooth corresponding test statistics. The effective degree of freedom (edf) is a proxy for the degree of non-linearity of the smooth. An edf of 1.0 indicates a linear relationship, while an edf > 1.0 indicates non-linearity. Edf.df = reference degree of freedom, Z = z-score, Chi.sq= chi-square, P = p-value, t = t-value, F = F-value.

| **Full sample**  **Only age** |  | **Horvath (N=2,223)** | | | |  | **Levine (N=2,223)** | | | |
| --- | --- | --- | --- | --- | --- | --- | --- | --- | --- | --- |
| **Δage** |  | **Estimate** | **SE** | **Z** | **P** |  | **Estimate** | **SE** | **Chi.sq** | **P** |
| (Intercept) |  | 1.78 | 0.11 | 16.59 | 2.38e-58 |  | -7.72 | 0.14 | -55.74 | 0.00e+00 |
| **Smooth terms** |  | **edf** | **edf.df** | **Chi.sq** | **P** |  | **edf** | **edf.df** | **Chi.sq** | **P** |
| s(Age) |  | 3.51 | 4.41 | 8.11 | 1.44e-06 |  | 3.16 | 3.97 | 10.21 | 0.00e+00 |
|  |  |  |  |  |  |  |  |  |  |  |
| **Full sample**  **Age x status** |  |  |  |  |  |  |  |  |  |  |
| **Δage** |  | **Estimate** | **SE** | **Z** | **P** |  | **Estimate** | **SE** | **Chi.sq** | **P** |
| (Intercept) |  | 0 | 0 | NA | NaN |  | 0 | 0 | NA | NaN |
| StatusSCZ |  | 1.4 | 0.28 | 4.91 | 1.00e-06 |  | -0.67 | 0.28 | -2.4 | 1.65e-02 |
| Dataset2009-086 |  | 0.22 | 0.66 | 0.33 | 7.40e-01 |  | -0.57 | 0.68 | -0.83 | 4.06e-01 |
| Dataset2011-023 |  | 2.03 | 0.89 | 2.28 | 2.25e-02 |  | -0.74 | 0.86 | -0.86 | 3.89e-01 |
| Dataset2013-234 |  | 0.37 | 0.66 | 0.56 | 5.74e-01 |  | -0.92 | 0.67 | -1.38 | 1.68e-01 |
| Dataset2015-9067 |  | -1.47 | 0.97 | -1.5 | 1.33e-01 |  | -1.26 | 1.06 | -1.18 | 2.38e-01 |
| DatasetGSE80417 |  | -8.47 | 0.31 | -27.71 | 9.44e-145 |  | 1.52 | 0.32 | 4.7 | 2.85e-06 |
| DatasetGSE84727 |  | -7.79 | 0.28 | -27.97 | 5.15e-147 |  | 0 | 0 | NA | NaN |
| Cohort-SCT |  | 0 | 0 | NA | NaN |  | 3.38 | 0.27 | 12.6 | 1.43e-34 |
| Cohort-NLD |  | -9 | 0.56 | -16.04 | 9.19e-55 |  | 2.48 | 0.61 | 4.09 | 4.51e-05 |
|  |  |  |  |  |  |  |  |  |  |  |
| **Smooth terms** |  | **edf** | **edf.df** | **Chi.sq** | **P** |  | **edf** | **edf.df** | **Chi.sq** | **P** |
| s(Age) |  | 1.8 | 2.52 | 0.5 | 6.85e-01 |  | 2.33 | 3.19 | 8.2 | 1.04e-05 |
| s(Age):StatusControl |  | 1 | 1 | 0.72 | 3.97e-01 |  | 1 | 1 | 0.02 | 8.92e-01 |
| s(Age):StatusSCZ |  | 1.46 | 1.78 | 2.66 | 8.02e-02 |  | 1 | 1 | 0.22 | 6.38e-01 |

**Table S12. GAM estimates of Δage trajectories across chronological age in men and women.** Shown are the statistical output of the GAM for Horvath (left) and Levine (right) Δage in men (top table) and in women (bottom table). The trajectory of Δage was modeled using a single smooth term of chronological age (in years) as predictors and a combined smooth term of chronological age and disease status. Shown are the estimate and standard error (SE) of the intercept and the fitted smooth corresponding test statistics. The effective degree of freedom (edf) is a proxy for the degree of non-linearity of the smooth. An edf of 1.0 indicates a linear relationship, while an edf > 1.0 indicates non-linearity. Edf.df = reference degree of freedom, Z = z-score, Chi.sq= chi-square, P = p-value, t = t-value, F = F-value.

|  |  | **Horvath** | | | |  | **Levine** | | | |
| --- | --- | --- | --- | --- | --- | --- | --- | --- | --- | --- |
| **Men (N=1459)** |  |  |  |  |  |  |  |  |  |  |
| **Δage** |  | **Estimate** | **SE** | **Z** | **P** |  | **Estimate** | **SE** | **Chi.sq** | **P** |
| (Intercept) |  | 0 | 0 | NA | NaN |  | 0 | 0 | NA | NaN |
| StatusSCZ |  | -0.67 | 0.28 | -2.4 | 1.65e-02 |  | 1.1 | 0.35 | 3.12 | 1.83e-03 |
| Dataset2009-086 |  | -0.57 | 0.68 | -0.83 | 4.06e-01 |  | -0.11 | 0.87 | -0.13 | 9.00e-01 |
| Dataset2011-023 |  | -0.74 | 0.86 | -0.86 | 3.89e-01 |  | 0.94 | 1.1 | 0.86 | 3.90e-01 |
| Dataset2013-234 |  | -0.92 | 0.67 | -1.38 | 1.68e-01 |  | 0.02 | 0.85 | 0.02 | 9.85e-01 |
| Dataset2015-9067 |  | -1.26 | 1.06 | -1.18 | 2.38e-01 |  | -2.12 | 1.35 | -1.58 | 1.15e-01 |
| DatasetGSE80417 |  | 1.52 | 0.32 | 4.7 | 2.85e-06 |  | -9.12 | 0.41 | -22.29 | 1.25e-94 |
| DatasetGSE84727 |  | 0 | 0 | NA | NaN |  | 0 | 0 | NA | NaN |
| Cohort-SCT |  | 3.38 | 0.27 | 12.6 | 1.43e-34 |  | -8.35 | 0.34 | -24.55 | 3.37e-111 |
| Cohort-NLD |  | 2.48 | 0.61 | 4.09 | 4.51e-05 |  | -8.77 | 0.77 | -11.39 | 7.61e-29 |
|  |  |  |  |  |  |  |  |  |  |  |
| **Smooth terms** |  | **edf** | **edf.df** | **Chi.sq** | **P** |  | **edf** | **edf.df** | **Chi.sq** | **P** |
| s(Age) |  | 2.33 | 3.19 | 8.2 | 1.04e-05 |  | 1.29 | 1.88 | 0.02 | 9.76e-01 |
| s(Age):StatusControl |  | 1 | 1 | 0.02 | 8.92e-01 |  | 1 | 1 | 1.9 | 1.69e-01 |
| s(Age):StatusSCZ |  | 1 | 1 | 0.22 | 6.38e-01 |  | 1 | 1 | 5.19 | 2.29e-02 |
|  |  |  |  |  |  |  |  |  |  |  |
|  |  |  |  |  |  |  |  |  |  |  |
| **Women (N=764)** |  |  |  |  |  |  |  |  |  |  |
| **Δage** |  | **Estimate** | **SE** | **Z** | **P** |  | **Estimate** | **SE** | **Chi.sq** | **P** |
| (Intercept) |  | 0 | 0 | NA | NaN |  | 0 | 0 | NA | 0 |
| StatusSCZ |  | -0.11 | 0.36 | -0.29 | 7.70e-01 |  | 2.22 | 0.5 | 4.46 | 2.22 |
| Dataset2009-086 |  | -0.98 | 0.73 | -1.34 | 1.80e-01 |  | 0.41 | 1.01 | 0.41 | 0.41 |
| Dataset2011-023 |  | 0.58 | 1.13 | 0.51 | 6.09e-01 |  | 4.2 | 1.57 | 2.68 | 4.2 |
| Dataset2013-234 |  | -1.17 | 0.83 | -1.42 | 1.56e-01 |  | 1.55 | 1.15 | 1.36 | 1.55 |
| Dataset2015-9067 |  | -0.38 | 1.01 | -0.37 | 7.08e-01 |  | -0.47 | 1.39 | -0.34 | -0.47 |
| DatasetGSE80417 |  | 0.52 | 0.33 | 1.59 | 1.13e-01 |  | -7.99 | 0.46 | -17.5 | -7.99 |
| DatasetGSE84727 |  | 2.99 | 0.38 | 7.92 | 9.42e-15 |  | -7.08 | 0.52 | -13.57 | -7.08 |
| Cohort-SCT |  | 0 | 0 | NA | NaN |  | 0 | 0 | NA | 0 |
| Cohort-NLD |  | 1.54 | 0.58 | 2.64 | 8.48e-03 |  | -9.53 | 0.81 | -11.77 | -9.53 |
|  |  |  |  |  |  |  |  |  |  |  |
| **Smooth terms** |  | **edf** | **edf.df** | **Chi.sq** | **P** |  | **edf** | **edf.df** | **Chi.sq** | **P** |
| s(Age) |  | 2.24 | 3.08 | 4.07 | 1.96e-02 |  | 1.79 | 2.51 | 2.14 | 2.63e-01 |
| s(Age):StatusControl |  | 1 | 1 | 0.04 | 8.42e-01 |  | 1 | 1 | 0.06 | 8.07e-01 |
| s(Age):StatusSCZ |  | 1 | 1 | 0.24 | 6.27e-01 |  | 1 | 1 | 1.46 | 2.27e-01 |
